# Supplementary material for: Polyacrylic Acid Functionalized Biomass-Derived Carbon Skeleton with Highly Porous Hierarchical Structures for Efficient Solid-Phase Microextraction of Volatile Halogenated Hydrocarbons
Source: Nanomaterials (Basel). 2022 Dec 8;12(24):4376. doi: 10.3390/nano12244376 (PMC9784554; doi:10.3390/nano12244376)
Supplement: Supplementary file 1 [file nanomaterials-12-04376-s001.zip › nanomaterials-2028398-supplementary.pdf]

nanomaterials

MDPI

Supplementary Information

Polyacrylic Acid Functionalized Biomass-Derived Carbon Skeleton with Highly Porous Hierarchical Structures for Efficient Solid-Phase Microextraction of Volatile Halogenated Hydro-carbons

Anying Long [1,2], Hailin Liu [3], Shengrui Xu [3,*], Suling Feng [3], Qin Shuai [4] and Shenghong Hu [1,*]

1 State Key Laboratory of Biogeology and Environmental Geology, School of Earth Sciences, China University of Geosciences, Wuhan 430074, China
2 113 Geological Brigade, Bureau of Geology and Mineral Exploration and Development Guizhou Province, Liupanshui 553000, China
3 Key Laboratory of Green Chemical Media and Reactions, Ministry of Education, Collaborative Innovation Center of Henan Province for Green Manufacturing of Fine Chemicals, School of Chemistry and Chemical Engineering, Henan Normal University, Xinxiang 453007, China
4 Faculty of Materials Science and Chemistry, China University of Geosciences, Wuhan 430074, China
* Correspondence: xushengrui@126.com (S.X.); shhu@cug.edu.cn (S.H.)

Section S1. Apparatus used for characterizations of materials

The microstructures of the PAA/N-SPCs were characterized by a scanning electron microscope at 5.0 kV with 10 mm working distance. The samples were prepared by adhering N-SPCs and PAA/N-SPCs powder onto carbon conductive tape. (SEM, SU8010, Hitachi, Tokyo, Japan). X-ray diffraction analysis was performed on a X-ray Powder diffractometer (XRD, X'Pert3 Powder, PANalytical B.V., Almelo, Holland, Cu K, λ=1.5418 Å) in a 2Θ range of 5–80° with scanning speed of 2° min^{-1} at 45 kV and 40 mA. Fourier-transform infrared (FTIR) spectra were recorded by a Thermo Nicolet NEXUS spectrometer (Thermo Fisher Scientific, Waltham, MA, USA) in the range of 4000–400 cm^{-1}. Before analyzed by FTIR, the samples were prepared by dispersing the materials in KBr pellets. The surface chemical groups were detected by X-ray photoelectron spectroscopy (XPS, VG Multilab 2000 X spectrometer, Thermo Fisher Scientific, Waltham, MA, USA) equipped with an Al Ka X-ray source (1486.6 eV). The energy step size of the XPS was 1 eV for survery scans and 0.05 eV for the fine scans. The BET surface area and pore size distribution were obtained by a nitrogen adsorption/desorption apparatus (Micromeritics ASAP 2020 M, Atlanta, GA, USA). The specific surface areas of the nanocomposite materials were determined based on the Brunauer - Emmett - Teller (BET) method through nitrogen adsorption at relative pressures ranging from 0.05 to 0.35 [1]. The condition of N_2 adsorption degassing was set at 200 °C for 6 h, and the mass of material analysed was 50.3 mg.

Reference

1. Singh, G.; Aher, S.C.; Varma, P.V.S.; Sathe, D.B.; Bhatt, R.B. Analysis of BET specific surface area in several recycled oxide powders. Journal of Radioanalytical and Nuclear Chemistry 2021, 327, 555-564.

Table S1. Operating parameters of of GC–MS

GC-MS injector	180 °C with splitless mode
Column type	HP-5 MS (30 m × 0.25 mm × 0.25 μm)
Flow rate of column	1 mL min^{-1}
Column oven	The initial temperature of 35 °C held for 2 min, then increased to 80 °C at the rate of 5 °C min^{-1} and held for 1 min.
MS Quad temperature	150 °C
MS source temperature	230 °C

Table S2. Retention time and characteristic ions of five VHCs.

Compounds	Retention (min)	Quantitative ions	Characteristic ions
$CHCl_3$	2.46	82.8	82.8、83.8
CCl_4	2.94	116.8	116.8、117.8
C_2HCl_3	3.52	131.8	59.9、94.7、131.8
C_2Cl_4	5.88	165.7	93.7、130.7、165.7
$CHBr_3$	8.13	172.6	171.6、172.6、173.6

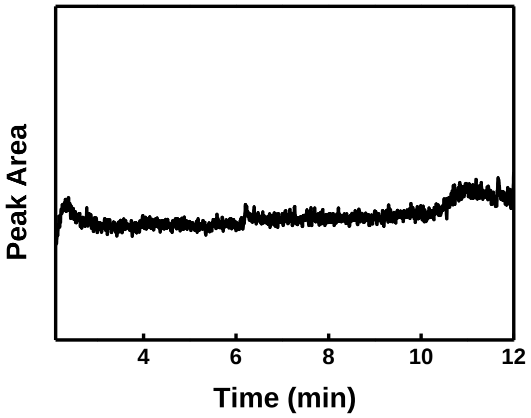

Figure S1. GC-MS chromatogram detected by a new PAA/N-SPCs fiber in the GC–MS injector at 250 °C.
